# ROS-Mediated Cancer Cell Killing through Dietary Phytochemicals

**DOI:** 10.1155/2019/9051542

**Published:** 2019-05-14

**Authors:** Saranya NavaneethaKrishnan, Jesusa L. Rosales, Ki-Young Lee

**Affiliations:** Department of Cell Biology and Anatomy, and Arnie Charbonneau Cancer and Alberta Children's Hospital Research Institutes, Cumming School of Medicine, University of Calgary, Calgary, Alberta, Canada T2N 4N1

## Abstract

Reactive oxygen species (ROS) promote carcinogenesis by inducing genetic mutations, activating oncogenes, and raising oxidative stress, which all influence cell proliferation, survival, and apoptosis. Cancer cells display redox imbalance due to increased ROS level compared to normal cells. This unique feature in cancer cells may, therefore, be exploited for targeted therapy. Over the past few decades, natural compounds have attracted attention as potential cancer therapies because of their ability to maintain cellular redox homeostasis with minimal toxicity. Preclinical studies show that bioactive dietary polyphenols exert antitumor effects by inducing ROS-mediated cytotoxicity in cancer cells. These bioactive compounds also regulate cell proliferation, survival, and apoptotic and antiapoptotic signalling pathways. In this review, we discuss (i) how ROS is generated and (ii) regulated and (iii) the cell signalling pathways affected by ROS. We also discuss (iv) the various dietary phytochemicals that have been implicated to have cancer therapeutic effects through their ROS-related functions.

## 1. Introduction

**Figure 1:**
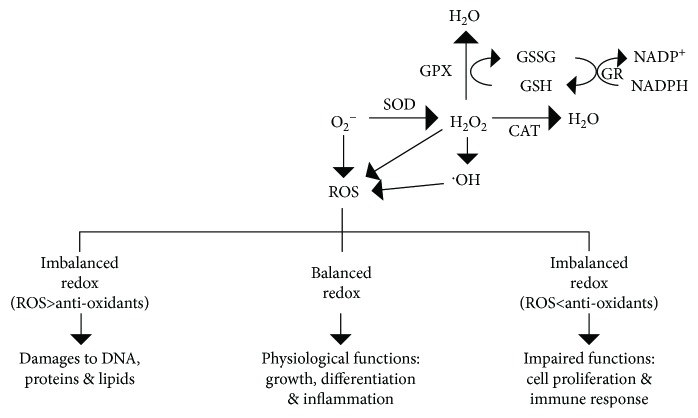
Intracellular redox homeostasis and imbalance and their effects on cellular functions. SOD: superoxide dismutase; CAT: catalase; OH: hydroxyl radical; GPX: glutathione peroxidase; GSSG: glutathione disulfide; GR: GSSG reductase; GSH: glutathione.

Reactive oxygen species (ROS) are highly reactive metabolic by-products that cause both deleterious and beneficial effects. Cellular ROS act as secondary messengers in signalling cascades that are critical for normal physiological functions such as differentiation and development [1, 2]. However, overproduction of ROS can cause damage to biomolecules such as DNA, lipids, carbohydrates, and proteins [3, 4], leading to loss of cell integrity and subsequently cell pathology (Figure 1). For example, ROS is now recognized to promote tumorigenesis, metastasis, and angiogenesis [5]. But then again, in cancer, excessive accumulation of ROS induces cell death [6]. Studies have shown that cancer cells have increased ROS level compared to normal cells due to high metabolic rate and mitochondrial dysfunction, which render increased susceptibility to oxidative stress [7, 8]. Thus, additional surge in ROS level is likely to cause cancer cells to reach their oxidative stress threshold sooner than normal cells, resulting in oxidative stress-induced cancer cell death [7, 8]. Therefore, it is not surprising that several natural dietary bioactive compounds that cause increased ROS levels have been shown to selectively target cancer cells [9]. For instance, dietary phytochemicals such as polyphenols, flavonoids, and stilbenes have the capacity to inhibit cancer cell proliferation and induce apoptosis and autophagy [10]. While most dietary bioactive compounds possess antioxidant capacity at low doses, high doses induce prooxidant activity that leads to cancer cell death. These compounds also influence mitochondrial functions by altering mitochondrial enzymes, oxidative phosphorylation, and mitochondrial pathways [11]. In this review, we focus on ROS regulation, ROS-mediated signalling pathways, and the contemporary use of dietary phytochemicals for cancer therapy.

## 2. ROS Regulation

**Figure 2:**
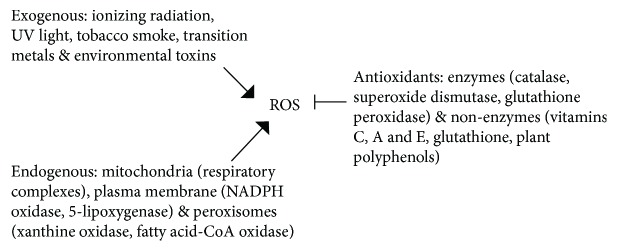
Exogenous and endogenous sources of ROS and enzymatic and nonenzymatic antioxidants.

ROS production is affected by both external factors such as tobacco smoke and ionizing radiation and intracellular factors such as the endoplasmic reticulum (ER), mitochondria, and peroxisomes [12] (Figure 2). Endogenous ROS are mainly produced in mitochondria during oxidative phosphorylation. Superoxide anions are generated through the electron transport chain complexes I and III localized in the inner mitochondrial membrane, and superoxide dismutase (SOD) converts superoxide ions into hydrogen peroxide (H_2_O_2_), which is subsequently catalyzed by glutathione peroxidase (GPX) to generate H_2_O. Catalase (CAT) also converts H_2_O_2_ to water (Figure 1) [13]. Other intracellular enzymes such as NADPH oxidase, lipoxygenases, and xanthine oxidase are also capable of ROS production [14]. Although intracellular redox homeostasis is well controlled by the enzymatic antioxidants, SOD, GPX, and CAT, it is also regulated by nonenzymatic antioxidants such as ascorbic acid (vitamin C) and glutathione (GSH) [15] (Figure 2).

Besides these antioxidants, the transcription factor, nuclear factor erythroid 2- (NFE2-) related factor 2 (Nrf2), also contributes in controlling oxidative stress. Activation of Nrf2 requires inhibition of its negative regulator Keap1, which results in Nrf2 nuclear translocation [16]. This leads to the expression and production of the antioxidant enzymes, CAT, GPX, heme oxygenase-1 (HO-1), and peroxiredoxin (PRX), and maintenance of redox balance [16]. We note, however, that intracellular oxidative stress induces activation of hypoxia-inducible factors (HIFs), resulting in the transcription of genes that promote survival and proliferation of cancer cells [17].

## 3. ROS in Cancer Signalling Pathways

ROS serve a crucial role in the regulation of a number of cellular processes such as cell proliferation and differentiation and cell death. Therefore, it is critical that a delicate balance in ROS level is maintained. ROS level is regulated by redox homeostasis via ROS elimination through antioxidants. Within the threshold limit of redox homeostasis, a regulated ROS increase could serve as a signal for H_2_O_2_-mediated oxidation of protein cysteine residues, triggering specific cellular events such as proliferation [18]. Conversely, disturbance of redox homeostasis in the direction of ROS overload leads to deleterious outcomes such as irreversible oxidative DNA damage that could trigger cell death. It is now known that metabolically transformed and fast-growing cancer cells have higher ROS levels than neighboring normal cells, placing cancer cells at a greater risk of reaching the ROS threshold to induce apoptosis. This infers that promoting further ROS production in cancer cells may be utilized as a strategy to induce cancer cell death.

**Figure 3:**
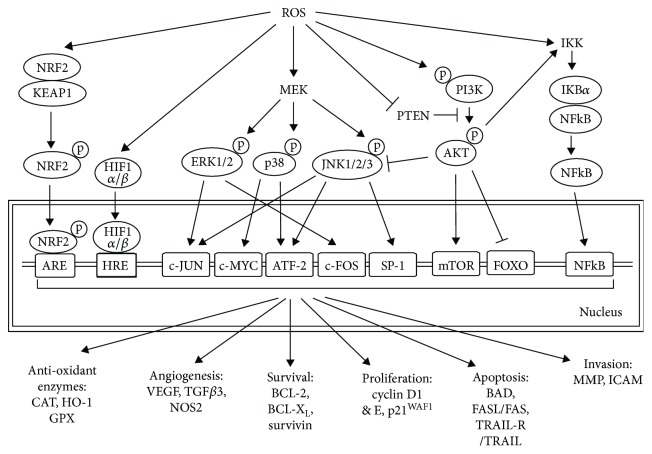
ROS-mediated intracellular cell signalling pathways. The indicated signalling pathways regulate molecules associated with angiogenesis, survival, proliferation, apoptosis, and invasion and the expression of antioxidant enzymes. NRF2: nuclear factor erythroid 2-related factor 2; KEAP1: Kelch-like ECH-associated protein 1; HIF1 *α*/*β*: hypoxia inducing factor 1 *α*/*β*; HRE: HIF-responsive elements; p38 MAPK: p38 mitogen-activated protein kinase; ERK: extracellular signal-related kinases; MEK: MAPK kinase; JNK; c-Jun N-terminal kinase; PTEN: phosphatase and tensin homolog; PI3K: phosphoinositide-3-kinase; AKT: protein kinase B; IKK: I*κ*B kinase; NF*κ*B: nuclear factor kappa-light-chain-enhancer of activated B cells; FOXO: forkhead box protein O; mTOR1: mechanistic target of rapamycin 1; ATF2: activating transcription factor 2; CAT: catalase; HO-1: heme oxygenase-1; GPX: glutathione peroxidase; VEGF: vascular endothelial growth factor; TGF*β*3: transforming growth factor beta 3; NOS2: nitric oxide synthase 2; BCL-2: B-cell lymphoma 2; BCL-X_L_: B-cell lymphoma-extra large; BAD: BCL2-associated agonist of cell death; TRAIL: TNF-related apoptosis-inducing ligand; MMP: matrix metalloproteinase; ICAM: intercellular adhesion molecule-1.

ROS play an important role in tumor initiation, promotion, and progression [19]. At levels below the ROS threshold, ROS activate oncogenes such as Ras and c-Myc [20] and induce p53-mediated DNA repair and survival [21] in cancer cells. At levels above the ROS threshold, ROS trigger apoptotic signals [6]. These cellular processes are controlled by ROS through its regulation of various signalling pathways (Figure 3), including the mitogen-activated protein kinase (MAPK)/extracellular-signal-regulated kinase (ERK), the phosphoinositide-3-kinase (PI3K)/protein kinase B (AKT), the inhibitor of kappa B (I*κ*B) kinase (IKK)/nuclear factor *κ*B (NF*κ*B), and the protein kinase D (PKD) signalling pathways [22, 23]. For example, ROS-dependent ERK activation controls the expression of proapoptotic genes by phosphorylation of transcription factors [23, 24]. Conversely, ROS-induced JNK activation results in phosphorylation and downregulation of antiapoptotic proteins such as BCL-2 and BCL-X_L_ [25]. In response to ROS, I*κ*B phosphorylation by IKK and subsequently ubiquitination lead to activation and translocation of NF*κ*B into the nucleus to stimulate the expression of antiapoptotic genes [26]. ROS directly activates PI3K subsequently converting phosphatidylinositol 4,5-bisphosphate (PIP_2_) to phosphatidylinositol 3,4,5-triphosphate (PIP_3_) and resulting in transcriptional inhibition of the AKT target genes, glycogen synthase kinase 3 (GSK3), forkhead box O (FOXO), and BCL-2-associated death promoter (BAD) and activation of mammalian target of rapamycin (mTOR1) [27].

**Figure 4:**
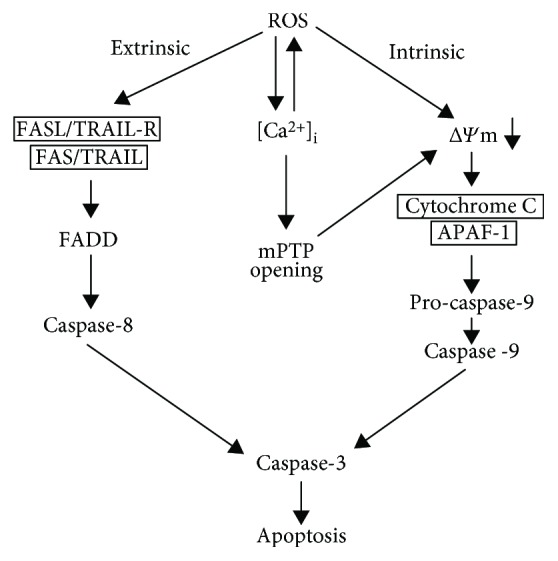
ROS-mediated extrinsic and intrinsic apoptotic pathways. TRAIL: TNF-related apoptosis-inducing ligand; FADD: Fas-associated death domain; [Ca^2+^]_i_: intracellular calcium concentration; mPTP: mitochondrial permeability transition pore; ΔΨm: mitochondrial membrane potential.

ROS-mediated apoptosis can be initiated by mitochondrial intrinsic apoptotic signalling or by extrinsic apoptotic signalling through death receptor pathways (Figure 4). Increased production of ROS depolarizes the mitochondrial membrane, releasing cytochrome C from the mitochondria. Cytochrome C induces activation of caspase-9 by promoting nucleotide binding to apoptotic protein-activating factor 1 (APAF-1), which leads to activation of caspase-3 [28]. Antiapoptotic (BCL-2 and BCL-X_L_) and proapoptotic (BAD, BAK, BAX, BID, and BIM) proteins also contribute to the formation of distinct channels for mitochondrial membrane permeabilization [29]. Elevated ROS levels have also been implicated in the activation of death receptors and in triggering caspase 8-mediated cleavage of caspase 3 [6]. In addition, ROS modulates the TRAIL- and Fas-mediated apoptosis through p53-mediated upregulation of death receptors. p53 regulates such apoptosis by controlling the expression of anti- and proapoptotic (e.g., PUMA and NOXA) proteins [30, 31]. ROS further promotes apoptosis by inducing increased Ca^2+^-mediated mitochondrial permeability transition pore opening [32].

## 4. Dietary Polyphenols

There is increasing claim that certain natural bioactive compounds can maintain redox homeostasis and hold promise as anticancer therapeutics due to their biocompatibility, biodegradability, comparatively less toxicity, and reduced side effects. The polyphenol bioactive compounds are secondary metabolites found in plants [33]. The most abundantly occurring plant polyphenols are phenolic acids and flavonoids which account for 30% and 60%, respectively, of dietary polyphenols [33]. Interestingly, they have both antioxidant and prooxidant properties that modulate cell proliferation and apoptotic pathways [34]. Some of the most common bioactive compounds that were suggested to have cancer therapeutic effects through their ROS-related activities are discussed below.

### 4.1. Quercetin

Quercetin (3,5,7,3′,4′-pentahydroxyflavone) is a flavonoid, present in numerous vegetables and fruits [34, 35]. Quercetin (Qu) displays neuroprotective, chemopreventive, and anticancer activities [36, 37], and these have been attributed to their anti- and prooxidative capacities. Qu efficiently scavenges mitochondrial superoxide anions (O_2_^−^) and subsequently generates semiquinone, Qu radicals, and H_2_O_2_ [11, 34, 38]. While, H_2_O_2_ is eliminated by peroxidase, semiquinone and Qu radicals alter intracellular ROS metabolism by depleting the intracellular GSH pool in a concentration-dependent manner [39–41] and inhibiting thioredoxin reductase activity [42]. *In vitro* and *in vivo* studies (Table 1) show that Qu promotes ROS-induced apoptosis, necrosis, and autophagy [43] at a range of 10-100 *μ*M in a variety of cancers, including glioma [43], osteosarcoma [44], and cervical [45] and breast cancer [46]. Qu induces apoptosis through distinct mechanisms: (i) via the mitochondrial pathway through activation of caspase-3. Qu reduces the mitochondrial membrane potential (MMP), inducing cytochrome C release and subsequent activation of caspase-3. This mechanism was observed in MDA MB-231 breast cancer cells [47], U937 promonocytic leukemia cells [48], HL-60 promyelocytic leukemia cells [49], HepG2 hepatocellular carcinoma cells [50], and oral cancer cells [51]. (ii) Qu alters the expression of the antiapoptotic BCL-2 and BCL-X_L_ and proapoptotic BAX and BAD proteins [47, 48]. Leukemic cells treated with Qu showed upregulation of BAX and increased phosphorylation of BCL-2 [52]. Similar results were observed in osteosarcoma [44] and breast cancer cells [46]. (iii) Qu induces the expression of death receptor- (DR-) 5, enhancing TNF-related apoptosis-inducing ligand- (TRAIL-) induced apoptosis [53–55] either by accumulating death receptors in lipid rafts [56] or inhibiting survivin in the ERK signalling pathway [57]. In addition to its proapoptotic capacity, Qu also promotes cell cycle arrest [58] by modulating p21*^WAF1^*, cyclin B, and p27*^KIP1^* in squamous cell carcinoma [59] and breast [60], lung [61], and hepatoma cancer cells [62].

### 4.2. Curcumin

Curcumin (1,7-bis(4-hydroxy-3-methoxyphenyl)-1,6-heptadiene-3,5-dione) is the principal polyphenol derived from turmeric (Curcuma longa). Various pharmacological activities have been attributed to curcumin, including its anti-inflammatory and anticarcinogenic properties which are triggered at 25 *μ*M [63]. Its anticancer effect is currently being evaluated in clinical trials for a variety of cancers [64–66] (Table 2). In normal cells, curcumin acts as a potent antioxidant. It scavenges hydroxyl radicals, superoxide, nitric oxide, H_2_O_2_, and peroxynitrite [11, 67–69] and modulates the expression of SOD, HO-1, and GPX through an indirect mechanism [11, 70–72]. In contrast, curcumin's anticancer properties rely on its prooxidative capacity to induce apoptosis, likely via the mitochondria-mediated pathway [73–75]. Curcumin oxidizes thiols in the mitochondrial membrane, leading to mitochondrial permeability transition pore (mPTP) opening, mitochondrial swelling, mitochondrial depolarization, and inhibition of ATP synthesis, resulting in apoptosis [76]. Evidence shows that curcumin increases ROS levels, including superoxides, hydroxy radicals, and H_2_O_2_ [77–79]. Indeed, in human hepatoma cells, curcumin causes cell death by ROS-induced mitochondrial DNA damage and impairment of OXPHOS [80, 81]. Curcumin also activates TRAIL-induced apoptosis by ROS-mediated upregulation of DR5 in renal cancer cells and colon cancer cells [82, 83]. Curcumin further induces autophagy in colon cancer cells through ROS-dependent activation of the ERK1/2 and the p38 MAPK pathway [84]. In glioblastoma [85] and liver cancer [86], curcumin decreases cancer stem cell viability and proliferation by ROS-mediated inhibition of NF*κ*B and signal transducer and activator of transcription 3 (STAT3). As with Qu, curcumin promotes cancer cell apoptosis by upregulating proapoptotic proteins (BAX, BIM, BAK, and NOXA) [87, 88] and downregulating antiapoptotic proteins (BCL-2 and BCL-X_L_) [89, 90]. In addition, curcumin can impede tumor angiogenesis by downregulating the expression of the vascular endothelial growth factor (VEGF) and matrix metalloproteinases (MMPs) [91, 92].

### 4.3. Capsaicin

Capsaicin (trans-8-methyl-N-vanillyl-6-nonenamide), the major component of Capsicum [93], has been implicated to have anticarcinogenic properties [94–96]. However, the mechanisms by which capsaicin induces cancer cell death are still unclear. The proposed anticancer mechanisms of capsaicin include promotion of ROS accumulation, mitochondria-mediated apoptosis, cell cycle arrest, and impairment of endoplasmic reticulum (ER) calcium homeostasis [97]. Capsaicin induces a rapid rise of ROS level followed by a disruption of mitochondrial membrane potential and subsequent activation of downstream caspase-3 in human colon cancer [98], pancreatic cancer [99], glioma [100], and prostate cancer [101]. In transformed T-cells, capsaicin inhibits the plasma membrane NADH-oxidoreductase (PMOR) electron transport chain, causing an increase in ROS level and subsequent disruption of the mitochondrial membrane potential [102]. Capsaicin at 150 *μ*M also blocks complexes I and III of the respiratory chain and decreases SOD activity in pancreatic cancer [103]. Interestingly, binding of capsaicin to the transient receptor potential vanilloid type 1 (TRPV1) results in an increase in intracellular calcium level and activation of the apoptotic pathway [104–106]. Besides its proapoptotic effects, capsaicin can also induce cell cycle arrest through inhibition of the cyclin-dependent kinases, Cdk2, Cdk4, and Cdk6 [107, 108].

### 4.4. Epigallocatechin-3-Gallate (EGCG)

Epigallocatechin-3-gallate ((2R,3R)-5,7-dihydroxy-2-(3,4,5-trihydroxyphenyl)-3,4-dihydro-2H-1-benzopyran-3-yl 3,4,5-trihydroxybenzoate) is a prominent catechin polyphenol in green tea. EGCG has dual antioxidant and prooxidant roles. It produces ROS by autooxidation [109] and its ability to modulate ROS level accounts for its chemopreventive property. EGCG induces apoptosis in various cancer cell types, including myeloid leukemia cells [110], human lymphoblastoid B cells [111], and hepatocarcinoma cells [112]. In pancreatic carcinoma [113] and lung cancer cells [114], EGCG-induced apoptosis occurs through inhibition of the PI3K/AKT signalling pathway. EGCG also decreases the mitochondrial membrane potential, increasing the intracellular free Ca^2+^ level and causing activation of the intrinsic apoptotic pathway. EGCG further decreases the expression of the antiapoptotic BCL-2, BCL-X_L_, xIAP, and cIAP and increases the expression of the proapoptotic BAD, BAX, and FAS/CD95 [115]. In pancreatic [116] and bladder cancer cells [117], EGCG also induces G_0_/G_1_ cell cycle arrest through regulation of cyclin D1, Cdk4, Cdk6, p21*^WAF1^*, and p27*^KIP1^* via the ERK, IKK, and PI3K signalling pathways. A combination of EGCG (10 *μ*M) and curcumin (10 *μ*M) inhibits breast cancer stem cell growth by inactivating the NF*κ*B-STAT3 pathway [118].

### 4.5. PEITC and BITC

Phenethyl isothiocyanate (PEITC) and benzyl isothiocyanate (BITC) are abundant in cruciferous vegetables that have been implicated to have anticancer properties [119–122]. Epidemiological studies show that increased intake of dietary isothiocyanates (ITC) reduces cancer risk [123] and increases cancer patient survival [124]. Both PEITC and BITC induce ROS production in many cancer cells [125–127]. IC_50_ value of PEITC is at the range of 3-14 *μ*M in various human cancer cells [128]. PEITC increases ROS level by decreasing intracellular GSH level, leading to mitochondrial dysfunction as observed in ovarian [126, 129] and non-small-cell lung cancer [128] cells but not in normal cells. PEITC-induced ROS production correlates with inhibition of complex III activity, inhibition of OXPHOS, and ATP depletion in prostate cancer [125]. PEITC also inhibits HO-1 and subsequently induces the ROS-mediated mitochondrial apoptotic pathway, which was noted in human chronic myeloid leukemia [130]. Conversely, BITC causes oxidative stress in pancreatic [131], glioma [122], and prostate cancer [132] cells by depleting SOD and GSH, which is accompanied by the induction of caspase-mediated apoptosis [121, 133]. BITC also activates the ERK/JNK/p38MAPK pathway in pancreatic cancer [134]. Both PEITC and BITC induce G_2_/M cell cycle arrest by downregulating cyclin B1, Cdc2, and Cdc25C [135, 136].

### 4.6. Piperine

Piperine ([5-(1,3-benzodioxol-5-yl)-1-oxo-2,4-pentadienyl]piperidine) is the most abundant natural alkaloid found in long pepper (Piper longum L.). Recently, it was determined to be a promising anticancer compound [137]. Piperine suppresses tumor growth in vitro and in vivo by modulating the ROS-induced oxidative stress response pathway, cell cycle arrest, and ER stress. In hepatocellular carcinoma, piperine treatment initiates ROS-induced mitochondria-mediated apoptosis by inhibiting catalase activity [138]. In human oral squamous cells exposed to high concentrations of piperine, ROS elevation is associated with mitochondrial depolarization and activation of caspase-mediated apoptosis. Piperine also induces nuclear condensation and cell cycle arrest in these cells [139].

### 4.7. Resveratrol

Resveratrol (3,4′,5-trihydroxystilbene), a polyphenol that is found in grapes and berries, effectively prevents tumor initiation and progression by stimulating apoptosis at 10 to 100 *μ*M [140] in prostate [141] and neuroblastoma cells [142]. Resveratrol has been shown to promote apoptosis by activating p53, ROS-dependent caspases, and death receptors for TRAIL and FasL [143]. Resveratrol-mediated apoptosis is mainly associated with the inhibition of the PI3K/AKT, MAPK, and NF*κ*B pathways [144] and STAT3 [145]. Moreover, resveratrol suppresses the expression of antiapoptotic proteins such as survivin, xIAP, and BCL-X_L_ and increases BAX/caspase-3-associated apoptosis [146]. Resveratrol further binds to F_1_-ATPase, inhibiting mitochondrial ATP synthesis [147, 148]. It triggers cell cycle arrest by upregulating p21*^WAF1^* and p27*^KIP1^* and downregulating cyclins D1, D2, and E and Cdks 2, 4, and 6 [149, 150].

### 4.8. Others

Peanuts, tomatoes, and carrots are rich in *p-Coumaric acid (p-CoA)*, an isomer of cinnamic acid [151]. In colon cancer cells, p-CoA triggers apoptosis by increasing ROS generation and mitochondrial depolarization, resulting in p53-mediated upregulation of BAX and downregulation of BCL-2 [151, 152]. In addition, p-CoA treatment of these cells in vitro and in vivo induces apoptosis mediated by the unfolded protein response [153].

The naturally occurring quinone compounds have potent cytotoxicity against cancer cells. In lung adenocarcinoma cells, *2-methoxy-1,4-naphthoquinone (MNQ) and 8-hydroxy-2-methoxy-1,4-naphthoquinone (HMNQ)* elicit ROS production and induce apoptosis via the JNK/p38 MAPK pathway [154–156].

*Naringenin*, a citrus flavonoid, triggers ROS-induced apoptosis and stimulates p38MAPK-mediated caspase activation [157, 158].

*Gallic acid (3,4,5-trihydroxy-benzoic acid; GA)*, which is widely present in grapes and red wine, inhibits lung cancer cell growth by increasing ROS level and depleting GSH [159]. In prostate cancer cells, autooxidation of GA produces H_2_O_2_ and O_2_^−^, leading to mitochondria-dependent apoptosis [160]. GA also induces apoptosis via ROS-dependent activation of the ATM/p53 [161] and JNK pathways [162].

## 5. Limitations

Poor bioavailability is a major obstacle for natural bioactive compounds, especially for Qu, curcumin, and resveratrol, which are associated with poor absorption and fast metabolism in the liver and intestine. Pharmacokinetic profile analysis of Qu revealed that about 93% of the compound is metabolised after oral administration (10 mg/kg) in male Sprague-Dawley rats [163]. On the other hand, people taking high oral doses (10 or 12 g) of curcumin attained limited availability of this compound in the plasma and other tissues [164]. Similarly, oral bioavailability of resveratrol is low at less than 1% [165]. Thus, the cytotoxic concentration of these compounds appears to be difficult to achieve by oral administration in cancer patients [166]. Several strategies have been proposed to overcome the problem of low oral bioavailability. One approach is to use a combination of phytochemicals. For example, a combination of piperine and curcumin [167] (in rats: 20 mg/kg piperine + 2 g/kg curcumin; in humans: 20 mg piperine + 2 g curcumin) or piperine and resveratrol [168] (in mice: 10 mg/kg piperine + 100 mg/kg resveratrol) showed increased bioavailability of curcumin and resveratrol, respectively. Other promising approaches include the use of novel formulations, synthetic analogues, prodrugs, and different drug delivery systems (e.g., via liposomes, phospholipid complexes, micelles, and nanoparticles). These methods could increase bioavailability as well as solubility and/or metabolic stability [169, 170]. Some studies have also shown that natural bioactive compounds may promote carcinogenesis by inducing ROS-mediated chromosome aberrations and DNA damage [80, 171, 172]. For example, an *in vivo* study showed that curcumin promotes lung cancer [173] and topical application of capsaicin causes skin cancer in mice [174], suggesting that these natural compounds must be carefully assessed for safety prior to clinical application.

As dietary phytochemicals lack mechanistic selectivity, these natural compounds display a variety of effects in different cancer cell types and thus the discrepancies in results among separate studies. Other possible reasons for divergent findings in different studies include changes or differences in (i) stability of the bioactive compounds in cell culture medium, for example, stability of Qu decreases at pH 7 or 8 [175]; (ii) release of bioactive compounds under different conditions, for example, the maximum release of curcumin occurs in phosphate buffered saline at pH 6.4 [176]; (iii) sensitivity of different cell types to bioactive compounds; (iv) cellular permeability of bioactive compounds; (v) presence or contamination by metal ions [177]; (vi) number of hydroxyl groups present in a molecule [177]; and (vii) *in vivo* biodistribution.

## 6. Conclusion

Natural phytochemicals have been associated with anticancer properties through their ability to modulate oxidative stress, cell cycle regulators, and proapoptotic, antiapoptotic, and survival signalling pathways. In preclinical and clinical trials, bioactive compounds show a promising and wide therapeutic window against various malignancies, including glioblastoma and breast, colon, and prostate cancers where phytochemical-induced cancer cell death was observed. However, certain attributes such as poor solubility and bioavailability of these bioactive compounds limit their clinical application. Thus, further studies are required to identify ways for effective biological delivery of these compounds in different cancer cell types. It is also critical that detailed studies are conducted in large cohorts to establish the pharmacokinetic profile of these compounds alone and in combination with other chemotherapeutic agents to determine dosage, tissue targets, and toxicity. Indeed, natural phytochemicals may serve as future therapy for specific types of cancer.

## Figures and Tables

**Table 1:** *In vivo* dosages and mechanistic effects of known natural bioactive compounds.

Compound	Animals	Cancer model	Dose	Mechanism
Quercetin	Male Sprague-Dawley rats	Glioma	100 mg/kg, every other day for 15 days, i.v	Autophagy and apoptosis [43]
	Female BALB/c mice	Colon & breast cancer	100 and 200 mg/kg for 36 days, i.p	Apoptosis [178]
	Male BALB/cA nude mice	Prostate cancer	20 mg/kg for 16 days, i.p	Antiangiogenesis [179]
	Female BALB/c/nude mice	Hepatic cancer	10 mg/kg for 7 days, i.p	Necrosis and antiproliferation [180]
	Female NOD.CB17-Prkdcscid/J lineage	Acute myelogenous leukemia	120 mg/kg, once every 4 days for 21 days, i.p	Apoptosis, autophagy, and cell cycle arrest [181]

Curcumin	Female BALB/c/nude mice	Colon cancer (multidrug resistance)	50 mg/kg, 2x/day for 14 days, peritumoral	Reduced expression of MDR1 and survivin [182]
	Male BALB/c/nude mice	Prostate cancer	25, 50, and 100 mg/kg, every 2 days for 30 days, abdominal cavity injection	Apoptosis [89]
	Female athymic nude mice	Breast cancer	45 mg/kg, 2x/week for 4 consecutive weeks, i.p	Antiproliferation [183]

Capsaicin	Female athymic nude mice	Pancreatic cancer	2.5 & 5 mg/kg, 5x/week, gavage	Activation of JNK and apoptosis [99]
	Female BALB/c nude mice	Colon cancer	1 & 3 mg/kg, 3 days once for 40 days, i.p	Apoptosis [95]
	Male BNX nu/nu mice	Prostate cancer	5 mg/kg, 3x/week for 4 weeks, gavage	Antiproliferation and apoptosis [184]
	Female BNX nu/nu	Breast cancer	5 mg/kg, 3x/week for 4 weeks, gavage	Reduced EGFR/HER2 activation and apoptosis [185]

ECGC	Female C3H/HeJ syngeneic mice	Squamous cell carcinoma	50 mg/kg, 5 days/week, i.p	Apoptosis [186]
	NOD/SCID mice	Myeloid leukemia	10 mM, oral drinking fluid	Antiproliferation [110]
	Female BALB/c mice	Bladder cancer	100 mg/kg for 4 weeks, i.p	Antiproliferation and migration [187]
	Male BALB/c/nude mice	Lung cancer	0.05% in drinking water for 21 days	Angiogenesis [188]
	Male BALB/c/nude mice	Adrenal pheochromocytoma	15 mg/kg, every other day for 15 days, i.p	Apoptosis [189]

PEITC	Male athymic nude mice	Glioblastoma	20 *μ*mol/100 *μ*l PBS for 21 days, gavage	Apoptosis [190]
	Male athymic mice	Prostate cancer	12 *μ*mol/100 *μ*l PBS for 5 days, oral	Apoptosis [191]
	Female BALB/c/nude mice	Lung cancer	25 mg/kg, 3x/week, i.p	Antiproliferation, reduced cancer stem cells [128]
	Female SCID/NOD mice	Breast cancer	81 mg/kg for 35 days, oral gavage	Apoptosis [192]
	Female athymic nude mice	Ovarian cancer	12 *μ*mol for 42 days, oral gavage	EGFR-AKT pathway inhibition, antiproliferation, and apoptosis [193]

Piperine	Female BALB/c mice	Mouse 4T1 mammary carcinoma	2.5 and 5 mg/kg, every 3 days for 3 times, intratumoral	Cell cycle arrest and apoptosis [194]
	Male nude mice	Prostate cancer	100 mg/kg/day for 1 month, i.p 10 mg/kg for 1 month, gavage	Antiproliferation and apoptosis [195]
	Male albino Wistar rats	Hepatocellular carcinoma (diethylnitrosamine-induced)	5 mg/kg, 3x/week for 6 weeks, oral	Apoptosis [138]

Resveratrol	Male nude mice	Lung cancer	20 mg/kg, every other day for 25 days, i.p	Reduce metastasis [196]
	Male BALB/c/nude mice	Bladder cancer	20 mg/kg/day for 4 weeks, i.p	Decreased VEGF and FGF-2 level, cell cycle arrest, and apoptosis [197]
	Female athymic mice	Breast cancer	25 mg/kg/day for 3 weeks, i.p	Apoptosis [198]
	BALB/c/nude mice	Pancreatic cancer	20, 40, and 60 mg/kg, 5 days/week for 6 weeks, gavage	Inhibition of FOXO transcription factors and apoptosis [199]
	Male athymic nude mice	Prostate cancer	50 mg/kg, every other day for 2 weeks, gavage	Antiproliferation [200]

i.p: intraperitoneal; i.v: intravenous.

**Table 2:** Clinical trials of natural phytochemicals.

Bioactive compounds (Clinicaltrials.gov identifier)	Disease condition	Phase	Dosage	Study goal
*Quercetin* (NCT03476330)	Squamous cell carcinoma	II	4 g/day	Efficacy in reducing buccal micronuclei in patients with Fanconi anemia
*Curcumin* (NCT03769766)	Prostate cancer	III	500 mg, 2x/day	Effect on prostate cancer progression
(NCT00094445)	Pancreatic cancer	II	8 g/day	Effect in pancreatic cancer growth and the safety of treatment
(NCT01246973)	Radiation dermatitis	III	500 mg, 3x/day	Effect on dermatitis caused by radiation therapy in breast cancer patients
With piperine (NCT02598726)	Neoplasms	I	A dose escalation study	Optimal biological dose in cancer patients
*Capsaicin* (NCT02037464)	Prostate cancer	II	2 capsules/day for 6 months	Expression of Ki67 and p27 in a posttreatment biopsy
(NCT00003610)	Head & neck cancer, mucositis	III	4 lozenges/day up to 2 weeks after radiation therapy	Efficacy of lozenges in patients with mucositis caused by radiation therapy
Patch (Qutenza) (NCT03317613)	Cancer	II	Qutenza (8% capsaicin patch) for every 3 months	Efficacy in peripheric neuropathic pain in cancer patients
*EGCG* (NCT02891538)	Colon cancer	Early I	450 mg, 2x/day	Chemopreventive effects
(NCT01317953)	Small cell lung carcinoma	I	2 × 450 mg/day to 5 × 450 mg/day	Side effects and best dose
*PEITC* (NCT00691132)	Lung cancer	II	4x/day for 5 days in week 4	Effect in preventing lung cancer in smokers
(NCT01790204)	Oral cancer	I & II		Effect on oral cells with mutant p53
Nutri-PEITC jelly (NCT03034603)	Head & neck neoplasms		200 mg/day, 5 days/week for 3 months	Safety and efficacy
*Resveratrol* (NCT00256334)	Colon cancer	I	20 mg/day	Modulation of Wnt signalling *in vivo*
(NCT01476592)	Neuroendocrine tumor		5 g/day	Effect on Notch-1 signalling
*SRT501* (NCT00920803)	Colorectal cancer	I	5 g/day	Safety and tolerability
